# The Development of a Green Innovative Bioactive Film for Industrial Application as a New Emerging Technology to Protect the Quality of Fruits

**DOI:** 10.3390/molecules27020486

**Published:** 2022-01-13

**Authors:** Waseem Ahmed, Rafia Azmat, Ebtihal Khojah, Rasheed Ahmed, Abdul Qayyum, Adnan Noor Shah, Asad Abbas, Sumeira Moin, Bassem N. Samra

**Affiliations:** 1 Department of Horticulture, The University of Haripur, Hatter Road, Haripur 22620, Pakistan; 2 Department of Chemistry, University of Karachi, Karachi 75270, Pakistan; rafiasaeed200@uok.edu.pk; 3 Department of Food Science and Nutrition, College of Science, Taif University, P.O. Box 11099, Taif 21944, Saudi Arabia; eykhojah@tu.edu.sa; 4 Department of Soil & Climate Science, The University of Haripur, Haripur 22620, Pakistan; rasheedahmad@uoh.edu.pk; 5 Department of Agronomy, The University of Haripur, Haripur 22620, Pakistan; aqayyum@uoh.edu.pk; 6 Department of Agricultural Engineering, Khwaja Fareed University of Engineering and Information Technology, Rahim Yar Khan 64200, Pakistan; ans.786@yahoo.com; 7 School of Horticulture, Anhui Agricultural University, Hefei 230036, China; asadabbas20088@yahoo.com; 8 Department of Botany, University of Karachi, Karachi 75270, Pakistan; sumeiramoin@hotmail.com; 9 Department of Biology, College of Science, Taif University, P.O. Box 11099, Taif 21944, Saudi Arabia; b.elsayed@tu.edu.sa

**Keywords:** apple, new bioactive films, *Melia azedarach* (Dharek), *Azadirachta indica* (Neem), bioactive compound, postharvest quality

## Abstract

Today, the most significant challenge encountered by food manufacturers is degradation in the food quality during storage, which is countered by expensive packing, which causes enormous monetary and environmental costs. Edible packaging is a potential alternative for protecting food quality and improving shelf life by delaying microbial growth and providing moisture and gas barrier properties. For the first time, the current article reports the preparation of the new films from Ditriterpenoids and Secomeliacins isolated from *Melia azedarach* (Dharek) *Azadirachta indica* plants to protect the quality of fruits. After evaluating these films, their mechanical, specific respirational, coating crystal elongation, elastic, water vapor transmission rate (WVTR), film thickness, and nanoindentation test properties are applied to apple fruit for several storage periods: 0, 3, 6, 9 days. The fruits were evaluated for postharvest quality by screening several essential phytochemical, physiological responses under film coating and storage conditions. It was observed that prepared films were highly active during storage periods. Coated fruits showed improved quality due to the protection of the film, which lowered the transmission rate and enhanced the diffusion rate, followed by an increase in the shelf life. The coating crystals were higher in Film-5 and lower activity in untreated films. It was observed that the application of films through dipping was a simple technique at a laboratory scale, whereas extrusion and spraying were preferred on a commercial scale. The phytochemicals screening of treated fruits during the storage period showed that a maximum of eight important bioactive compounds were present in fruits after the treatment of films. It was resolved that new active films (1–5) were helpful in the effective maintenance of fruit quality and all essential compounds during storage periods. It was concluded that these films could be helpful for fruits growers and the processing industry to maintain fruit quality during the storage period as a new emerging technology.

## 1. Introduction

The chemical and physical processes of liquid drying films have a universal history in engineering and protection facilities with appealing requirements and barriers. The active compounds forming thin-film recently gained attention as having advanced chemical, photographic, and electrical properties [1,2]. It forms a dry film, remains non-hazardous, and is desirable to provide reliable performance in all applications [3]. Natural films are primarily attractive to food industries for food safety, controlling diseases, and maintaining food quality under specific environmental processes. The films have the potential of several cations, minerals, specific compounds, and molecules to maintain food quality.

Apple (*Malus domestica B*), the world’s most popular fruit for its health-related elements and nutritional value, belongs to the *Pomoideae* subfamily *Rosaceae*. It is ranked third after banana and citrus for worldwide consumption and uses. It was reported that the losses during postharvest, packaging, transportation, handling, storage, and marketing of fresh apples occur by 20–40% [2]. The most common factors that affect the quality of apples during storage and after harvesting; include respiration, water loss, microbial activities, and metabolism changes [3,4,5]. The fruit industry always tries to manage the quality and shelf life during the storage of fruits and vegetables [6]. Numerous phytonutrient losses reported in fruits/vegetables are greatly affected by the duration of storage [7]. Postharvest treatments maintain fruit quality and freshness; considerable differences in physiological parameters such as ethylene production in fruits and respirational changes have been noted in previous reports [8,9]. Fruit quality parameters such as the fruit texture and water loss are linked through maturity and harvesting times [10]. Active films or coated materials play a vital role in fruit perseveration and safety during storage [7]. The internal and external quality conditions of apple fruits linked with the postharvest losses directly affect fruit quality parameters such as total soluble contents, firmness of fruit, juice contents, fruit weight losses, and pH [11,12]. Many advantageous techniques have been developed to control storage quality, which can retain fruit quality to keep them in good condition for a more extended period. Coated materials at postharvest storage were applied in the form of film to achieve the desired appearance, resulting in inhibition of losses that also satisfy the consumers [13]. It helps in delaying fruit softening and upsurges the fruit’s firmness; besides, it also delays the physiological disorder and increases the shelf life of many vegetables and fruits [5,14]. At the same time, the use of chemicals to preserve food is toxic to human health during storage [14,15]. *A.indica* (Neem) is a rich source of several bioactive compounds with large chemical diversity, which can be used after isolation [5,14]. The extracted compounds of *A. indica* (Neem) belong to two groups triterpenoids, and diterpenoids secomeliacins, such as salanin, azadirachtin non-isoprenoids, coumarin tannins, and dihydrochalcone, and also aliphatic compounds [16] showed a vast range of biological activities which were safe to maintain the quality of fruit and its preservation process during storage period [17,18].

This article deliberated the preparation of new bioactive films for the first time through Ditriterpenoids and Secomeliacins isolated from *Melia azedarach* (Dharek) and *Azadirachta indica* (Neem) plants. After preparing new bioactive films, thickness and nanoindentation tests were also applied for the first time. The films after preparation process and protocol development were checked against mechanical, respirational, coating crystal, elongation, elastic nodule, and water vapor transmission rate (WVTR) of the apple fruits and stored to measure their quality, phytochemical screening, quality enzymes, taste-related compounds, and physiological responses of fruits under storage and film application. It was established that newly developed films might be used to preserve food, pharmaceutical products, and chemical industry as future enactment of products coated at the upper surface, such as in this research where apples were used for testing the prepared films and found effective.

## 2. Material and Methods

### 2.1. Isolation and Purification of Novel Bioactive Compounds from Leaves of Melia azedarach (Dharek) and Azadirachta indica for Films Preparation

**Figure 1:**
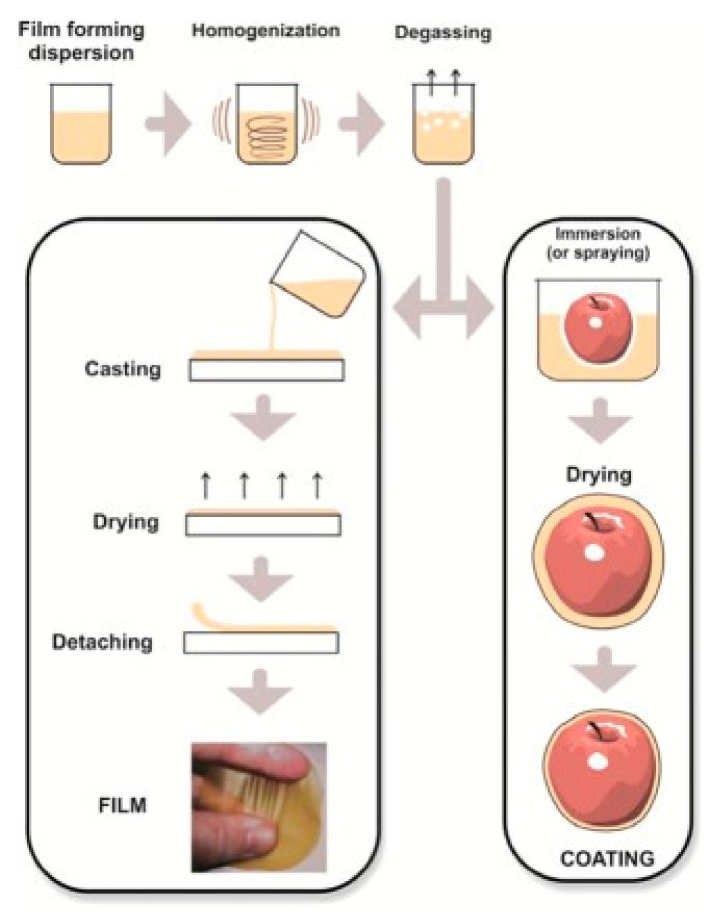
A graphical representation of the development of the films and application process.

The samples of (Dharek and Neem) leaves of both plants were dried at 72 °C temperature, then ground to get dry leaves powder; after that, different concentrations of liquid solutions were prepared with the help of the filtration process, as shown in (Table 1). The two targeted compounds of Ditriterpenoids and Secomeliacins were obtained from (Dharek and Neem) leaves extracts. Design details of novel films are mentioned in (Table 1), and the development process is illustrated in Figure 1.

### 2.2. Films Preparation

Five pure films were prepared from organic coated materials made by two significant compounds like Ditriterpenoids and Secomeliacins. The compounds were in liquid form and obtained in (3 mL) with adding of 90% of deacetylation, Aqua Premier Co., Bang Lamung/Chon Buri, Thailand and 1% of lactic acid (Acros Organics, Geel, Belgium) in solutions added and stirred for 10 min at room temperature until the compounds of Dtriterpenoids + Secomeliacins were dissolved entirely in solutions. The glycerol at 1% (*w*/*v*) (Panreac, Madrid, Spain) and Tween 80 at 0.2% (*w*/*v*) (Acros Organics, Belarus Belgium) were added for making a plasticizer form and as a surfactant, respectively. The pH 5.5 of the films was noted.

### 2.3. Standardization of Diterpenoids and Secomeliacins Compounds in Films Uses

The solutions of diterpenoids and secomeliacins were obtained from two medicinal plants *Melia azedarach* (Dharek) and *Azadirachta indica* (Neem) were standardized. The pure solution of both compounds was used in a concentration of 1–3% based on dryness. The prepared films were cast in Petri plate for the drying process. They were stored at 20°C (controlled by laboratory air conditioning system) and 53% RH (obtained by equilibration in a desiccator with a saturated salt solution of Mg(NO_3_)_2_, under vacuum), until further use as a proper film (Figure 1).

### 2.4. Protocol of Novel Films Developmental Process

The films of binding agents (Diterpenoids + Secomeliacins) were prepared in pure solution. Film making and shaping created a film structure and foaming pattern with conventional scattering or suspension. Mixtures of 10–30 mg of diterpenoids + secomeliacins—blended to make homogeneous concentration solutions and lowered endeavoring of mixtures to obtain complete films.

#### 2.4.1. Degassing of Films

The degassing and defoaming of manufactured films is a significant advancement in creating new films of diterpenoids + secomeliacins to eliminate air microbubbles in solutions left in suspended films, entangled the films, and deformities that were mechanically matched to its structure. The plasticized films of 2 mL of corn syrup were used for vacuum degassing films with the evacuation of 30 m bar films sizes in 4 min.

#### 2.4.2. Bench Casting Process

The films were created by seat projecting on the rimmed or Palin plates for the manufacturing process. The film thickness was measured in the rimmed plates, drawdown bars, or plain plates (Table 2). The standard cards solution of film shaping was achieved in the seat projecting process. The surface of the film structure was checked in drying and measured at the temperature or in airflow boilers at temperatures not higher than 30 to 40 °C, for 12 to 48 h.

#### 2.4.3. Thickness of Films

The thickness of each film was measured using a digital micrometer (Mitutoyo, MFG, Kawasaki, Japan). For each produced sample, six determinations were made at random positions.

The water vapor permeability measurement in active films to its potential and suitability for the food industry was conducted (Table 2).

The method of Bertuzzi et al. [19] was used to determine water vapor permeability in active films. The conditioned films were sealed to a glass dish containing distilled water using silicone adhesive to give good seal formation. A saturated sulphuric acid solution was placed on the glass dish in a desiccator maintained at 25 °C and 52% relative humidity. The water vapors transferred through the films were determined by measuring the weight changes periodically until a constant weight was reached for about 6 h. The water vapor permeability was calculated from the slope of the linear regression of weight loss versus time. The formula is given below:

Water vapor permeability (WVP) was calculated from the following equation:

WVP = CXAΔP(1)

where X is the film thickness (m), A is the area of the exposed film (m^2^), ∆P is the water vapor pressure differential across the film (Pa), and C is the slope of the weight gain of the dish, to the 0.0001 g, versus time.

The temperature dependency of WVP of the edible films was evaluated using an Arrhenius relationship (Equation (2)) described by Bertuzzi et al. [19].

WVP = WVPoexp(−EP/RT)(2)

where WVP is the water vapor permeability coefficient, WVP is a constant, EP is the activation energy (J/mol) of permeation, R is the universal gas constant 8.314 J/mol. K and T is the absolute temperature (Kelvin). Logarithmic transformation of the above equation gave:

From the slope of the fitted regression line, the apparent activation energy (EP) of films was determined at test temperatures.

The gravimetrically ASTM E96-92, 1990 determined the water vapor permeability (WVP) as a reported method of Cerqueira et al. [20]. The WVP was measured using the formula reported by Sobral et al. [21]:

Wx × WAPx t(3)

where x is the average thickness of edible films, A is the permeation area (0.005524 m^2^). DP is the difference of partial vapor pressure of the atmosphere (2337 Pa at 20 °C) and was the weight loss. Two replicates were obtained for each samples.

### 2.5. Mechanical Properties of Novel Films

Tensile strength (TS) and duration during breaks (EB) remaining EAEP films were measured by SUNS 6102 Universal Testing Machine (Shenzhen, China) according to standard ASTM D882-12 (Table 2). The films were cut into strips 13 mm and 170 mm long and placed at a temperature of 25 °C and 50% RH for 48 h before measuring. A loading cell of 100 N was used to detect sample properties. The initial catch distance was 100 mm, and the crosshead speed was 5 mm/min. The reported values were an average of eight ratings for each film. The parameters observed in this study include the characteristics and small structure of the edible film. The film thickness was measured using the micrometer screw model MDC-25M (MFG, Kawasaki, Japan) with an accuracy of 0.001 mm. The flexibility during the break was measured using the Universal Instrument Tensile Strength Meter (ASTM D882-1). The film was cut at 10 × 5 cm and stretched at a 50 mm/min speed. Elongation was then measured according to the formula:

Description:

L = length of edible film during breaks (mm)

L0 = original length (mm)

### 2.6. Film Microstructure

The structure of the film was detected by scanning electron microscopy (Scanning Electron Microscopes , (SEM) JEOL Ltd. JSM 5310 LV Peabody, Bostan, MA, USA). The film was cut to 0.5 × 0.5 cm^2^, then placed on a carbon-coated plate and covered with gold. The sample was then placed on an SEM device for small structure monitoring.

### 2.7. Stretch Modulus and Nanoindentation Process in Films

Two mechanical features, modulus and elasticity, and stiffness were determined by nanoindentation. The elastic modulus (E), commonly referred to as Young’s modulus is a measure of stress (soku) pressure (ε) when the twist is fully stretched. In an expansive region, stress and difficulty are proportional to Hooke’s rule: σ = Eε.

Hardness films (H) measure resistance to material in conversion by inclusion. The transformation of plastic is caused by the movement of the separation of the atomic structure of an object.

### 2.8. Moisture Contents and Water Solubility of Films

Approximately 50 mg of the film were dried at 104 °C for 24 h (until the equilibrium weight was attained). The weight loss was determined, and MC was calculated as the percentage of water removed from the system. The film solubility in water was determined by the reported method [22].

### 2.9. Collection of Fruits for Trials Process

The samples of delicious golden apples were collected from Haripur, Khyber Pakhtunkhwa, Pakistan. The trials were conducted in the Department of Horticulture laboratory, The University of Haripur, and other chemical processes were analyzed in the Department of Chemistry University of Karachi, Pakistan.

### 2.10. Washing and Grading of Apple

The apples (Golden) were washed with sodium hydrochloride (1PPM) solution added in one liter of solution. The apples were dipped in the solution, washed after ten minutes, dried, and graded based on their proper size and shape.

### 2.11. Application of Films to an Apple Fruit

The film of proper concentration on each apple was applied for 15 min after washing and grading, simply by dipping in solution to cover the entire apple surface. The apples were placed for 3 min for complete dryness of the bioactive wax thickness, then shifted into a plastic basket and placed at room temperature for 9 days storage with 3-day intervals. In each replication, 5 apples were selected.

### 2.12. Stored Apples at Room Temperature

The apples were treated with specific coating materials, used as a source of treatments, and stored at room temperature, where the initial temperature was 32 °C and humidity was 70%. The storage periods were 3, 6, and 9 days. The days after storage was compared with untreated fruits.

### 2.13. Films Effects on Physiological Rates and Oxidative Stress in Stored Apples

The MDA and the Lipid peroxidation contents were determined by the thiobarbituric acid (TBA-MDA) reaction as reported by Hodges et al. [23], Ali et al. [24]. The Chlorophyll contents of apples were measured by the method [25]. The Li and K Flux membrane stability (mg dm^−3^) was measured by the method [26].

### 2.14. Quality Standards of (Golden) Apples during Storage Periods with Response to New Films

#### 2.14.1. Total Soluble Solids (TSS)

The total soluble solids were measured with the help of advanced refractometer 1.2 BX-CV, New York, NY, USA.

#### 2.14.2. Total Phenolic Contents (TPC)

Total phenolic contents (TPC) were measured using the Folin-Ciocalteu assay according to the procedure described by Stintzing et al. [27] with slight modifications. The Folin-Ciocalteu reagent (1 mL) was diluted five times and was mixed with 0.2 mL of sample and 0.8 mL 7.5% Na_2_CO_3_. The reaction was performed for 20 min at room temperature in darkness, and the absorption was recorded at 765 nm against the control sample. The results were expressed as mg equivalent of gallic acid (GAE) per gram according to the calibration curve built in the range of 0.02–0.10 mg gallic acid used as a standard.

#### 2.14.3. Determination of DPPH Radicals Scavenging Activity

The DPPH radicals scavenging activity was estimated with the method used by Williams et al. [28] with some modification, 4 mg DPPH was dissolved in 100 mL methanol and stored in a dark place. Different concentrations (50, 100, and 150 µL) were mixed with 5 mL of 0.004% DPPH solution and left in sunlight. The absorbance was measured at 517 nm after 20 min of reaction against the corresponding blank solution. The assay was performed in triplicates.

Percentage inhibition of free radical DPPH was calculated based on control reading by the following (Equation (4)):

Equation (4) = DPPH scavenged (%) = (A con − A test) × 100

A con

A con—is the absorbance of the control reaction

A test—is the absorbance in the presence of the sample of the extracts.

### 2.15. Quality Enzymes of (Golden) Apples after Films Application

#### 2.15.1. Superoxide Dismutase (SOD)

The activity of superoxide dismutase (SOD) was determined according to Das et al. [29] with slight modification. Fifty microliters of the enzyme extract were mixed with a reaction mixture (500 µL of phosphate buffer of pH 7.4, 200 µL methionine, 200 µL triton, 100 µL NBT and 500 µL of 50 mM EDTA) and left in sunlight for 15 min and then 100 µL of riboflavin added. The absorbance of the sample was recorded at 560 nm.

#### 2.15.2. Catalase Assay (CAT)

The catalase activity was determined according to Wheeler et al.’s [30] method with slight modification. The 100 µL enzyme extract was added to the reaction mixture containing 100 µL of 5.9 mM H_2_O_2_ and 800 uL of 0.01 M phosphate buffer (pH 7.0), and the absorbance was measured at 240 nm. Time taken for a decrease in the absorbance from 0.45 to 0.4 was noted. The enzyme activity was expressed as a mole of H_2_O_2_ consumed/min/mg protein.

#### 2.15.3. Peroxidase Assay (POX)

The Peroxidase (POX) activity was determined according to Bergmeyer et al. [31] with slight modification. The reaction mixture consisted of 100 µL of 40 mM H_2_O_2_, 100 µL 20 mM Guaiacol, and 800 µL 0.1 M phosphate buffer pH 7.0. 100 µL enzyme extract was added to 100 µL of the reaction mixture, and the absorbance was recorded at 470 nm.

### 2.16. Screening of Essential Phytochemicals of Apples

During the storage period, several essential phytochemicals were measured in the apple juice; the saponins test was measured in 2 mL of each sample where 5 mL of distal water was added and well shaken for 30 s, the stable frothing persistent indicate that saponins are present in it. The tannin test was measured into 2 mL of each sample and heated up to 40 °C, then add 5–6 drops of FeCl_3_ added, light green coloration, shows the presence of tannin. The steroids were measured by mixing 2 mL of the sample with 1 mL of H_2_SO_4;_ the red layer showed a presence of steroids in the solution. The terpenoids were measured in the juice of apple by adding 2 mL of each sample with adding of 2 mL of chloroform and 3 mL H_2_SO_4_, reddish-brown coloration the presence of terpenoids. The phlorotannins were measured in apple juice with 2 mL of juice sample then 1 mL of HCl was added. The presence of red color indicates the presence of phlorotannins. The cardiac glycoside was measured by adding 1 mL of glacial acetic acid into 2 mL of each sample, and 2–3 drops of FeCl_3_ show green-blue coloration reflecting the presence of cardiac glycosides. The flavonoids were measured by mixing 2 mL of each sample into 2 mL of NaOH and then adding 1 mL of HCl shows a yellow color, and their disappearance shows flavonoids in apple juice. The quinines were measured in 2 mL of each sample, then added 1 mL of NaOH, which resulted in blue-green or red color indicating the presence of quinines.

### 2.17. Specification of HPLC-DAD Methods

The HPLC machine is linked with a detector (DAD) with a frequent wavelength of 500–3000 milli-absorbance units. The HPLC system used a (Milford, MA, USA) binary water pump attached to a photodiode array detector. The system attached an auto-injector with a water phase analytical column. The mobile phase was checked through ultrasonic waves before the operation. The ultrasonic machine was attached with a digital timer and temperature-controlled power of 400–900 W with a frequency of 45 Khz.

#### 2.17.1. Analysis of Chromatographic Conditions for Bioactive Compounds under Film Coating

The mobile phase composition was optimized for best resolution results with a short analysis time. The major bioactive compounds were isolated by suppressing the acidic mobile phase. The acidic mobile was best for a longer retention time, and a better peak shape was obtained. Diverse mobile phase compositions, such as (methanol, 0.1% aqueous ethylic acid *v*/*v*, acetonitrile, and aqueous 0.1% *v*/*v* 0.1% formic acid and methanol) were evaluated. Formic acid and methanol 0.1% were best for the mobile phase as all components resolved under this condition. The five compounds were separated by elution of isocratic mode, the eluation mode was followed by (A) formic acid 0.1 *v*/*v* and (B) methanol for separation a liner program is followed 10–65% B 0–85 min, and the standard mixture should be baseline separated for each chosen marker.

#### 2.17.2. Analysis of Major New Bioactive Compounds to Taste of Apple

HPLC was performed with a Varian-Agilent system, consisting of a ternary pump 9050 series and a UV–Vis detector 9050 series-coupled to STAR 4.5 software for data acquisition and elaboration. A Gemini NX C18 110 À column, 250 × 4.6 mm (Phenomenex), protected by a guard column, was at the stationary phase. The five active compounds and two essential compounds under bioactive films were separated by gradient elution with 12% (*v*/*v*) formic acid in water (solvent A) and 40:40:20 (*v*/*v*/*v*) acetonitrile: water:formic acid (solvent B), according to the gradient. The injection volume was 20 μL, and the column temperature was 20 °C, whereas the detection length was 260 nm. Before injection in the HPLC system, samples were filtered through a PTFE 0.45 μm pore-size filter. Quantification was performed based on external standards of known concentrations.

**Statistical Data Analysis**

The obtained data were subjected to statistical analyses using statistical software Statistics 8.1 by applying the CRD design in this research. The significance of the data for further mean calculations (LSD) least significant difference, which gives us mean lettering of the data, was measured. The graph was prepared by the software Graph Pad Prism 7.02. The HPLC-DAD measured the new taste compounds by Chrom gate v 3.31 Knauer software.

**Results and Discussions**

**Comparison of Tensile strength (N), elongation at break (EB), Thickness (mm) Elongation (%), and water vapor transmission rate (WVTR) changes in new films**

**Figure 2:**
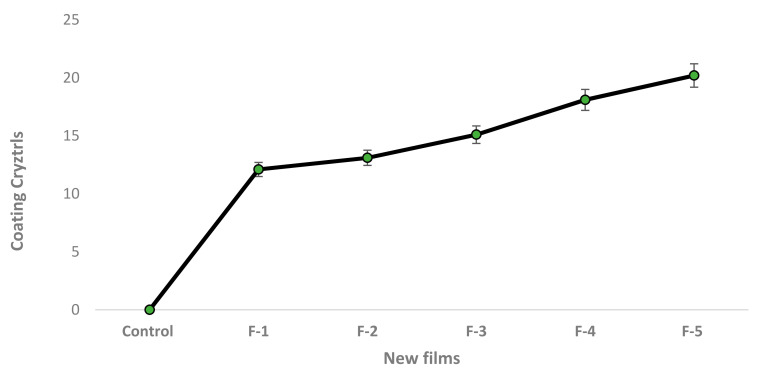
Changes in coating crystal and thickness in films.

Significant differences were found in tensile strength and films activity. Results showed that both compounds’ higher activity was successfully used in edible films. The analysis of variance (Table 2) showed that the addition of compounds in different concentrations impacted the elongation of the edible film. The elongation at break thickness and elongation (%) is higher in Film-5. Film elongation increases the strength of the film. It is noticed that films with lower water vapor transmission rate (WVTR) and transmission rate significantly enhanced the shelf life of products. The coating crystal was higher in Film-5, and lower activity is noticed in untreated films Figure 2.

**Films properties with stability, activity, and potential applications**

**Figure 3:**
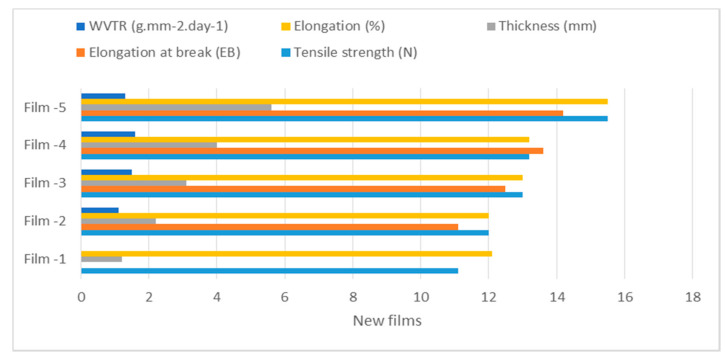
Mechanical and structural changes in films.

**Figure 4:**
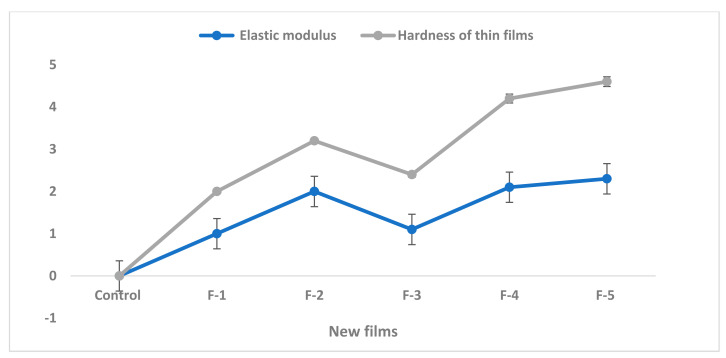
Elasticmodulus and hardness of new films.

**Figure 5:**
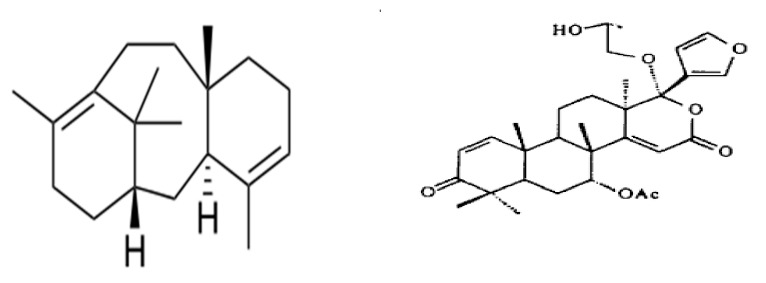
Structural formula of two essential compounds for preparation of films and its developmental process.

The new films of (diterpenoids + secomeliacins) were applied to the apple surface before the storage period and accounted for water vapor permeability (WVP), thermal stability, and film stability. Initial values (12.1, 13.2, 16.4) were recorded at 0 days, as shown in (Table 3, Figure 3 and Figure 4). However, slight changes were noticed at 3, 6, and 9 days of storage periods (Table 3). All treated fruits showed significant results in the film stability process, whereas the film 5 performed better than the control fruits. Lower film activity was noted in all three selected parameters in control. The elastic strength and hardness of films were higher in films-4 and 5, and lower activity was observed in untreated fruits shown in Figure 3. The films stability used in the apple was 14–16% shown in Figure 4 and Figure 5.

**Respirational changes in apples during storage periods under a coating of new films**

**Figure 6:**
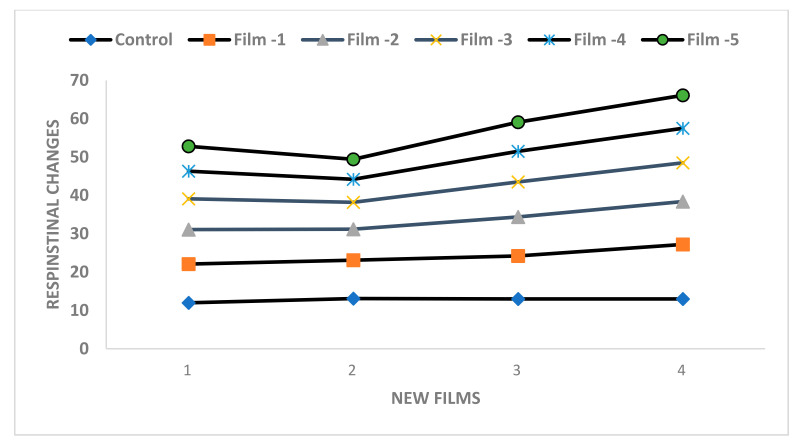
Respirational changes in apple fruits at storage periods with new films.

The comparison of storage period and application of new films on respiration changes of apple fruits were measured and shown in Table 4. The higher oxygen, CO_2_, ethylene, and respirational rates were (12, 13, 13.4, and 16.4) were noticed at 0 days, whereas at the others’ storage, like 3, 6, and 9 days showed lower rates shown in Figure 6. Generally, the films’ application on apple reduced the respirational rates as noticed for Film-5. Higher rates of oxygen, CO_2_, ethylene, and respiration were noted, in the untreated fruits.

**Quality enzymatic changes and storage periods of apples (golden delicious)**

Statistically significant differences were found regarding apple treatment and storage interactions by applying bioactive films (Ditriterpenoids and Secomeliacins) (Table 5). Slight changes were noticed at 3-, 6-, and 9days storage. The control Film-1 showed a higher range of CAT (6394.7) for 6 days of storage. The treated fruit and untreated fruit showed no significant difference in POX. Superoxide dismutase (SOD) showed a significant difference during the storage period, while treatment interaction the highest value is of Film-5 on day 9 on which all combined treatments (combine treatments of 50 mg Dharek and Dharek/200 mL distal water 20 mg Ditriterpenoids and Secomeliacins, 20 mL bioactive compounds of in solutions) were applied, and the lowest value is of Film-4 on day 6. The storage was continued for 9 days. Pox was observed higher on day 9 stored fruits than on days 3 and 6 stored fruits. The activity of SOD showed a significant increase on the 9th day storage with the value (SOD = 790.9 μ/g).

**Bioactive compounds of apple fruits under films coating**

Significant differences were found in treated fruits of apples stored at ambient temperature. The higher value of TSS was noticed for Film-4 and Film-5 shown in Table 6. The untreated fruits showed a reduction in TSS, while in storage; this trend was with the days extended. The total highest antioxidants TA (46.411%) was noticed in the treatment of T2. However, the lower values of TA (36.01%) were noticed for untreated fruits. The higher value of TPC noticed is (0.1863%), while the lower value of TPC (0.16%) is noticed in untreated fruit after 9 DAS.

**Screening of phytochemicals of apples under new films**

The screening of phytochemicals in (Golden Delicious) during storage periods of (3, 6, and 9days) shown in Table 7 where eight essential phytochemicals such as saponins, tannins, steroids, terpenoids, phlobatannins, flavonoids, and quinines are reported. All stored fruits showed rich contents of eight essential phytochemicals during the screening process. These were isolated from the pulp of the apple. It was observed that Films 3 showed higher values of cardiac glycosides in apple fruits at 9 days of storage, while at 3 and 6 days, stored fruit showed poor contents of cardiac glycosides in apple fruits. The results of this study support that apple fruit pulp is a rich source of phytochemicals.

**Taste related essential bioactive compounds of stored apples under film coating**

**Figure 7:**
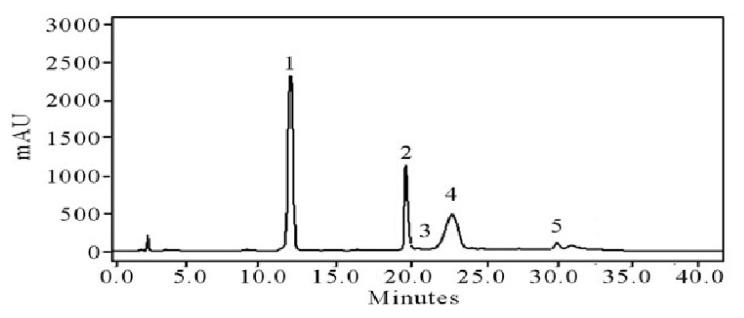
HPLC-DAD chromatogram of major taste of quality compounds of apple with numerical peak (1–5) phloridzin, chlorogenic acid, flavonoids, feurlic acid, P-coumaroylquinic.

The five essential bioactive compounds were evaluated in fresh and stored golden delicious apples, as shown in Table 8 and Figure 7. The essential five active compounds were first-time isolated from the pulp of apple (phloridzin, chlorogenic acid, flavonoids, ferulic acid, P-coumaroylquinic) through the HPLC-DAD method. The comparison of stored and treated fruits for these five essential active compounds was conducted. These compounds were isolated at 0, 3, 6, and 9 days; stored fruits showed slight variations in these compounds at 9 days. The noticed range of phloridzin, chlorogenic acid, flavonoids, ferulic acid, P-Coumaroylquinic was 10.0, 12.1, 15.2, 10.5, and 16.0, respectively. The higher value was noticed at 9 days of storage. A higher range of all five essential compounds was measured in all treated fruits. For the treatment of T5 30 mg (Ditriterpenoids and Secomeliacins) films, a higher range of all five important bioactive compounds was noted. Comparing stored fruits and treated fruit showed a clear difference in essential bioactive compounds. Similar is with the taste compounds chromatogram shown in Figure 7.

**Physiological changes in stored apples under films coating**

The physiological parameters showed significant differences between treatments and storage periods of fruits shown in Table 9. The stored fruit showed specific changes in their physiological responses such as malondialdehyde (MDA), chlorophyll contents (%), lipid peroxidation assay per (g) FW Li and K contents, flux membrane stability (mg dm), and improved the shelf life of apples. The 3-day stored fruits showed low variations while 9-day stored fruits showed a more considerable change in these specific parameters. In comparison, treated fruit showed the higher maintained all specific parameters, in Table 7.

## 3. Discussion

Apples are a highly nutritional crop globally [32], and their quality is directly linked to storage periods. It is challenging to maintain the quality of apples without treatments in postharvest storage life [33]. The excess use of chemicals and reagents has been frequently performed to maintain the postharvest quality of the fruit. Still, this excessive use causes toxic effects on fruits, the environment, and human health [34]. Researchers are now focused on the organic and safe prevention methods of fruits and vegetables being more protuberant to the postharvest method [35]. The fruits and food products are highly perishable, requiring additional protection from decay and spoilage during their distribution, preparation, and storage [34]. The use of organic extracts and powerful bioactive compounds isolations can improve and maintain the postharvest life of apples during storage periods [34]. The results reported in Table 1 and Table 2 showed that higher enzymatic activity and phytochemicals were observed in stored, treated fruits with coated films [35]. The film maintained the fruit quality and improved the shelf life [34] followed by safe and healthy apple fruits during storage [32]. The fruit softening and quality enzymes are a responsible factor for limiting the storage life of horticultural crops; the application of the proper film on apples reduced deleterious changes in metabolism and cell wall structural response during the storage period; the films protected the fruits from losses and saved the signal induction pathways; the quality and TSS were increased during the storage period due to dehydration and hydrolysis of polysaccharides. The bioactive films can be attributed to their roles in lowering the respiration rate and delaying the conversion of starch into simple sugars and other impacts, such as decreasing the weight loss and ethylene biosynthesis, hence delaying the ripening process [36]. Polyphenolic acid and color compounds are major health-related compounds of apples and were attained at 9 days of storage. No previous reports are available regarding the effect of these treatments either applied as a post or pre-harvest treatment on screening phytochemical’s color compounds and physiological responses at oxidative stress in storage periods. The detailed literature search showed that the neem and Dharek extracts have potential for controlling decays, water loss, and maintaining fruit quality during the storage periods, as reported by Bajpai et al. [32], Laciar et al. [37], and Asadujjaman et al. [36]. The bioactive films can control postharvest disease and improve the shelf life of apples [36]. The effects of organic treatments improve the shelf life and quality of apples, as mentioned in studies by Bajpai et al. [32] and Asadujjaman et al. [36]. Previous studies reported the alteration in enzyme activities related to changes in metabolites by applying organic extracts Davarynejad et al. [38]. (2013); Nasira et al. [39]. The current results are in accordance with the reports of [36,40], where the increased postharvest quality of apples was observed under the application of leaves extracts of Neem. Similar results were reported in neem formulation/extracts in mango and apples reported earlier by [35,40]. The phytochemicals in Neem and dharek showed diterpenoids + secomeliacins compounds that increased apple quality and shelf life during the storage period [36]. The role of diterpenoids + secomeliacins extracts plays a vital role against the microbial activity. It helps in inhibiting the respiration rate of fruits and the transpiration of fruits [41]. It was reported that several leaf and organic extracts were applied in fruits crops of apples, mango, and cheery fruits [37]. The results of the current study are in agreement with those that explain the effects of the natural extracts on enzymes changes and modified antioxidants activity [38,42]. The results explained the effects of the combined treatment of natural extracts and pure organic compounds to improve the shelf life and quality of apples during storage periods.

## 4. Conclusions

The greatest challenge encountered by food manufacturers is the loss of quality of food products during storage, which eventually adds to significant wastage and losses. This study used the preparation of new films for the first time with added active compounds Ditriterpenoids and Secomeliacins. The prepared films were tested for their mechanical, specific respirational, and coating crystal elongation, elastic and water vapor transmission rate (WVTR), film thickness, and nanoindentation. Tests were carried out for new films to assess the impact on apple’s storage periods, quality, and shelf life (Golden Delicious). The five new active films were used in this study with 10–30 mg concentration ranges. The treated fruits of apples with coated films attained maximum fruit quality compared to untreated fruits. Eight proper initial screening tests of phytochemicals were conducted with treated and untreated fruits. Higher values were noted in treated fruits of apples with higher ranges than uncoated fruits. The taste-related and color formation bioactive compounds of (phloridzin, chlorogenic acid, flavonoids, ferulic acid, p-coumaroylquinic) isolated in apple pulp through the advanced and suitable method of HPLC-DAD. All essential compounds were saved under films coating during storage periods, and the physiological response under oxidative stress of films stability parameters was also better in all treated fruits. Based on the results, the films help to prevent diseases and maintain fruit quality during storage. The commercial storage life can be extended by applying films or coated materials for fruits industries.

## Figures and Tables

**Table 1:** The design of new five films developed.

Novel Films	
Film-1	10 mg of Diterpenoids + Secomeliacins film per fruit
Film-2	15 mg of Diterpenoids + Secomeliacins film per fruit
Film-3	20 mg of Diterpenoids + Secomeliacins film per fruit
Film-4	25 mg of Diterpenoids + Secomeliacins film per fruit
Film-5	30 mg of Diterpenoids + Secomeliacins film per fruite

Films (1–5) novel compounds of Diterpenoids + Secomeliacins.

**Table 2:** Mechanical properties of the new films.

New Film Specific Parameters	Film-1	Film-2	Film-3	Film-4	Film-5
Tensile strength (N)	11.1 ± 0.81	12.0 ± 0.80	13.0 ± 0.19	13.2 ±0.19	15.5± 0.19
Elongation at break (EB)	10.0 ± 0.89	11.1 ± 0.89	12.5 ± 0.89	13.6 ± 0.89	14.23 ± 0.29
Thickness (mm)	1.2 ± 0.89	2.2 ± 0.89	3.1 ± 0.89	4.0 ± 0.89	5.6 ± 0.83
Elongation (%)	12.1 ± 0.89	12.0 ± 0.89	13.0 ± 0.89	13.2 ± 0.89	15.5 ± 0.19
WVTR (g·mm^−2^·day^−1^)	2.0 ± 0.89	1.1 ± 0.89	1.5 ± 0.89	1.6 ± 0.89	1.3 ± 0.86

Mean of three determinations ± SD of films.

**Table 3:** Comparison of film stability parameters.

Film Specific Parameters	Day after Storage (DAS)					Application of Films				
	0	3	6	9	Control	Film-1	Film-2	Film-3	Film-4	Film-5
Water vapor permeability (WVP)	12 ^a^	12.0 ^a^	11.5 ^b^	10.0 ^c^	10.0 ^c^	12.1 ^b^	12.0 ^b^	13.0 ^e^	13.2 ^b^	15.5 ^a^
Thermal stability	13.2 ^a^	13.1 ^a^	12 ^b^	12.1 ^c^	9.00 ^f^	10.00 ^e^	10.5 ^d^	11.5 ^c^	11.6 ^b^	11.23 ^a^
Films stability (mm) (df > 50)	16.4 ^a^	16 ^b^	15.5 ^c^	15.2 ^d^	13.00 ^e^	14.2 ^b^	13.2 ^c^	13.1 ^d^	13.00 ^e^	14.6 ^a^

Values are presented as the mean ± 95% confidence; values followed by different superscript letters (^a–f^) are significantly different at (*p* < 0.05).

**Table 4:** Comparisons (DAS and new films) on respirational changes in apple fruits.

New Film Specific Parameters	Days after Storage (DAS)				Film Application					
	0	3	6	9	Control	Film-1	Film-2	Film-3	Film-4	Film-5
Oxygen	12 ^a^	11.0 ^b^	10.5 ^c^	9.0 ^d^	12.0 ^a^	10.1 ^b^	9.0 ^c^	8.01 ^d^	7.2 ^e^	6.5 ^f^
Co2	13.2 ^a^	11.1 ^b^	10 ^c^	8.2 ^d^	13.1 ^a^	10.0 ^b^	8.1 ^b,c^	7.0 ^d^	6.01 ^e^	5.2 ^f^
Ethylene	13.4 ^a^	10.2 ^b^	9.5 ^c^	8.2 ^d^	13.0 ^a^	11.2 ^b^	10.2 ^c^	9.1 ^d^	8.00 ^e^	7.6 ^f^
Respirational rate	16. 4 ^a^	12.2 ^b^	11.5 ^c^	10.2 ^d^	13.0 ^a^	14.2 ^b^	11.2 ^c^	10.1 ^d^	9.00 ^e^	8.6 ^f^

Values followed by different superscript letters (^a–f^) are significantly different (*p* < 0.05); values are presented as the mean ± 95% confidence.

**Table 5:** Effects of new films on quality enzymes of catalase (CAT) (μ/g) Peroxidase (POX) (μ/g) superoxide dismutase (SOD) (μ/g) (Golden Delicious).

Quality Enzymes Changes of Stored Fruits and Films									
New Prepared Films	Catalase (CAT) (μ/g)			Per-Oxidase (POX) (μ/g)			Superoxide Dismutase (SOD) (μ/g)		
	DAS 3	DAS 6	DAS 9	DAS 3	DAS 6	DAS 9	DAS 3	DAS 6	DAS 9
Control	4692.0 ^abcdef^	4997.3 ^abcde^	4398.1 ^abcdef^	6939 ns	11,981 ns	14,906 ns	527.61 ^f^	638.74 ^cdef^	597.21 ^def^
Film-1	3623.7 ^bcdef^	6394.7 ^a^	5547.3 ^abc^	6310 ns	8454 ns	9557 ns	548.26 ^ef^	543.83 ^ef^	684.82 ^abcd^
Film-2	3634.5 ^bcdef^	2658.6 ^f^	4381.3 ^abcdef^	6572 ns	7665 ns	6235 ns	544.90 ^ef^	522.41 ^f^	659.77 ^bcde^
Film-3	3272.0 ^def^	4454.2 ^abcdef^	5229.2 ^abcd^	5819 ns	5546 ns	10,191 ns	544.56 ^ef^	638.64 ^cdef^	730.14 ^abc^
Film-4	4297.9 ^abcdef^	3458.8 ^cdef^	3861.6 ^bcdef^	6386 ns	4498 ns	6804 ns	543.57 ^ef^	517.67 ^f^	773.44 ^ab^
Film-5	2902.5 ^ef^	4600.9 ^abcdef^	5691.6 ^ab^	8071 ns	4928 ns	7399 ns	560.14 ^def^	780.94 ^ab^	790.70 ^a^

Values are presented as the mean ± 95% confidence; values followed by different superscript letters (^a–f^) are significantly different at (*p* < 0.05).

**Table 6:** Effects of new films on total soluble solids, total antioxidants, and total phenolic compounds (Golden Delicious).

New Prepared Films	Total Soluble Solid (TSS)			Total Antioxdants % Inhabitation of DPHH			Total Phenolic Compounds mg/100 g		
	DAS 3	DAS 6	DAS 9	DAS 3	DAS 6	DAS 9	DAS 3	DAS 6	DAS 9
Control	3.7000 ^a^	3.4000 ^ab^	2.3667 ^cd^	30.640 ^cde^	35.709 ^b^	46.769 ^a^	0.1720 ^abc^	0.1596 ^cd^	0.1637 ^bcd^
Film-1	2.5333 ^bcd^	2.7333 ^abcd^	2.3667 ^cd^	34.480 ^bc^	44.260 ^a^	43.441 ^a^	0.1613 ^bcd^	0.1521 ^d^	0.1571 ^cd^
Film-2	2.5000 ^bcd^	2.6667 ^bcd^	2.5000 ^bcd^	36.017 ^b^	26.493 ^e^	46.411 ^a^	0.1634 ^bcd^	0.1581 ^cd^	0.1605 ^cd^
Film-3	2.9667 ^abc^	2.5333 ^bcd^	2.5667 ^bcd^	33.712 ^bc^	27.978 ^de^	45.387 ^a^	0.1595 ^cd^	0.1639 ^bcd^	0.1624 ^bcd^
Film-4	2.1667 ^cd^	1.7667 ^d^	2.1667 ^cd^	34.736 ^bc^	32.381 ^bcd^	34.480 ^bc^	0.1687 ^bcd^	0.1783 ^ab^	0.1863 ^a^
Film-5	2.4667 ^bcd^	2.0667 ^cd^	2.0667 ^cd^	35.044 ^bc^	30.691 ^cde^	33.354 ^bc^	0.1546 ^d^	0.1557 ^cd^	0.1659 ^bcd^

Mean sharing same letter in row or column showed significant difference at 5% probability level (LSD) and Ns showed a non-significant, 10, 15, 20, 25, and 30 mg of diterpenoids + secomeliacins bio compound used in films developmental process.

**Table 7:** Qualitative screening of phytochemical screening in apples (Golden Delicious).

DAS	New Films on Screening of Phytochemical in Apples (Golden Delicious)								
	Films	Saponins	Tanin	Sterols	Terperoids	Phlobatannins	Cardic Glycoside	Flvonoids	Quinines
3	Control	+	+	+	+	+	−	+	+
3	Film-1	+	+	+	+	+	−	+	+
3	Film-2	+	+	+	+	+	−	+	+
3	Film-3	+	+	+	+	+	−	+	+
3	Film-4	+	+	+	+	+	−	+	+
3	Film-5	+	+	+	+	+	−	+	+
6	Control	+	+	+	+	+	−	+	+
6	Film-1	+	+	+	+	+	−	+	+
6	Film-2	+	+	+	+	+	−	+	+
6	Film-3	+	+	+	+	+	−	+	+
6	Film-4	+	+	+	+	+	−	+	+
6	Film-5	+	+	+	+	+	−	+	+
9	Control	+	+	+	+	+	−	+	+
9	Film-1	+	+	+	+	+	−	+	+
9	Film-2	+	+	+	+	+	−	+	+
9	Film-3	+	+	+	+	+	+	+	+
9	Film-4	+	+	+	+	+	+	+	+
9	Film-5	+	+	+	+	+	+	+	+

(−): Negative test (absence of turbidity, flocculation, and precipitation); (+): Test strongly positive (if the reagent produces a precipitate or flocculation heavy).

**Table 8:** Comparisons (DAS and treated fruits) of new and taste related major bioactive compounds isolated from pulp of (Golden Delicious) using HPLC-DAD methods.

Major Taste Compounds of Apples (u/g) 100 g	Day after Storage Changes				New Films Application of Apples					
	0	3	6	9	Control	Film-1	Film-2	Film-3	Film-4	Film-5
Phloridzin	12.10 ± 0.80	12.00 ± 0.85	11.50 ± 0.81	10.00 ± 0.81	10.00 ± 0.84	12.10 ± 0.81	12.00 ± 0.81	11.00 ± 0.81	12.10 ± 0.81	12.50 ± 0.81
Chlorogenic acid	13.20 ± 0.81	13.10 ± 0.81	12.50 ± 0.81	12.10 ± 0.81	9.00 ± 0.21	10.00 ± 0.81	10.50 ± 0.81	11.50 ± 0.81	11.60 ± 0.81	11.23 ± 0.81
Flavonoids	16.40 ± 0.81	16.00 ± 0.80	15.50 ± 0.81	15.20 ± 0.81	13.00 ± 0.812	14.20 ± 0.81	13.20 ± 0.81	11.50 ± 0.81	13.00 ± 0.81	14.60 ± 0.81
Feurlic acid	12.50 ± 0.12	11.50 ± 0.80	11.10 ± 0.81	10.50 ± 0.81	11.00 ± 0.12	12.10 ± 0.81	12.30 ± 0.81	12.50 ± 0.81	13.10 ± 0.81	13.40 ± 0.81
P-Coumaroylquinic	18.10 ± 0.823	18.00 ± 0.14	17.00 ± 0.81	16.00 ± 0.81	16.00 ± 0.81	16.00 ± 0.81	16.50 ± 0.81	14.00 ± 0.81	15.00 ± 0.83	17.00 ± 0.81

Mean values of stored fruit and treated fruits were measured.

**Table 9:** Comparisons (DAS and new films) of physiological parameters of films.

Physiological Parameters	Day after Storage (DAS)				Treated of New Films					
	0	3	6	9	Control	Film-1	Film-2	Film-3	Film-4	Film-5
Malondialdehyde (MDA) contents per gram FW	12.1 ^a^	12.0 ^a^	11.5 ^b^	10.0 ^c^	10.0 ^d^	11.1 ^c^	12.2 ^b^	11.0 ^c^	12.3 ^b^	12.5 ^a^
Chrophyll contents in leaves (%)	13.2 ^a^	13.1 ^a^	12.5 ^b^	12.1 ^c^	9.00 ^f^	10.00 ^e^	10.5 ^d^	11.5 ^c^	11.6 ^b^	11.23 ^a^
Lipid peroxidation assay per gram FW	16.4 ^a^	16 ^b^	15.2 ^c^	15 ^d^	13.00 ^e^	14.2 ^b^	13.2 ^c^	13.1 ^d^	13.0 ^e^	14.6 ^a^
Li and K	12.0 ^a^	11.0 ^b^	10.5 ^c^	10 ^d^	11.00 ^f^	12.1 ^e^	12.3 ^d^	12.5 ^c^	13.1 ^b^	13.4 ^a^

Values followed by different superscript letters (^a–f^) are significantly different (*p* < 0.05); values are presented as the mean ± 95% confidence.

## Data Availability

No data is available publically can be provided on suitable request.

## Sample Availability

Samples of the compounds are stored in the repository of University of Haripur, for intented future experimentation, and can be obtained from corresponding author upon suitable request.

## Data Available

The data that support the findings of this study are available from the corresponding author upon reasonable request.

## Article Classification

Chemical syntheses/processing, Mechanical/Physical processing, Processing.

## Engineering Industrial Suitability

New active packaging films developed in this study with natural extracts compounds obtained from plants and active agents (prepared by natural extraction) have shown the potential to improve the stability of films and their proper activity and managements, process, the new technology is support to chemical and pharmacy other industry uses as a preservative of raw materials, properties with advances of mechanical, chemical uses as future of the industrial application.
